# Reference interval for immature platelet fraction on Sysmex XN haematology analyser in adult population

**DOI:** 10.11613/BM.2018.010708

**Published:** 2018-02-15

**Authors:** Claudia E. Imperiali, Ariadna Arbiol-Roca, Lourdes Sanchez-Navarro, Macarena Dastis-Arias, Juan C. Lopez-Delgado, Anna Cortes-Bosch, Ana Sancho-Cerro, Dolors Dot-Bach

**Affiliations:** 1 Clinical Laboratory, Hospital Universitari de Bellvitge, L’Hospitalet de Llobregat, Barcelona, Spain; 2 Critical Care Unit, Hospital Universitari de Bellvitge, L’Hospitalet de Llobregat, Barcelona, Spain

**Keywords:** platelets, haematology, reference values, immature platelet fraction

## Abstract

**Introduction:**

The Sysmex XN-series haematology analyser has newly adopted a fluorescent channel to measure immature platelet fraction (IPF). To promote the clinical utility of this promising parameter, establishing a reliable reference interval is mandatory. According to previous studies, IPF values may be affected by the employed analyser and the ethnic background of the individual, but no differences seem to be found between individuals’ genders. Therefore, this study aimed to define the reference interval for IPF in a Spanish population following Clinical and Laboratory Standard Institute (CLSI) guidelines.

**Materials and methods:**

A total of 153 healthy Caucasian adults from Spain met the inclusion criteria. IPF measurement was performed by means of a Sysmex XN-2000 haematology analyser. A non-parametric percentile method was used to calculate the reference intervals in accordance with CLSI guidelines.

**Results:**

The obtained reference interval for IPF on the Sysmex XN-2000 was 1.6–9.6% (90% confidence intervals (CIs) were 1.5–1.8 and 9.3–11.5, respectively). No significant gender difference in IPF reference intervals was observed (P = 0.101).

**Conclusions:**

This study provides, for the first time, a reference interval for IPF using a Sysmex XN-2000 in a Spanish population, ranging from 1.6 to 9.6%. These data are needed to evaluate platelet production in several conditions such as thrombocytopenia, inflammatory states and cardiovascular diseases, as well as for future research.

## Introduction

In the last decades, automated haematology analysers have undergone important technological upgrades, such as the introduction of new methods and channels, the optimization of reagents and an improvement in analysis algorithms. This has resulted in an optimization of the analytical characteristics and in an increase in the obtained information. However, this additional information and the potential clinical uses of the new parameters have not been widely studied. This is the case with immature platelet fraction (IPF), which is a new parameter that measures young or immature platelets in peripheral blood. This parameter is usually expressed as a proportional value of the total platelet count (%-IPF), as well as absolute immature platelet (A-IPF), which is a derived value from the total platelet count and %-IPF.

Immature platelets are the newly released platelets from the bone marrow by megakaryocytes. They have a short lifespan (< 24 hours) (*1*). The IPF is a helpful and non-invasive biomarker for the diagnosis of thrombocytopenia. It makes a distinction between central failures and peripheral platelet destruction because it is an indicator of megakariopoetic activity (*2*, *3*). Bone marrow activity is low in patients with central thrombocytopenia, so the IPF would be low. In contrast, in conditions where there is peripheral platelet destruction the bone marrow activity is accelerated and the IPF would be high (*2*).

Immature platelet fraction can also be an early predictor of bone marrow recovery after chemotherapy and stem cell transplantation (*4*, *5*). Generally, IPF values increase several days prior to a rise in the platelet count avoiding unnecessary platelet transfusions (*6*).

In recent years, several potential uses of IPF have been reported in the literature related to cardiovascular outcomes and inflammatory conditions (*7*-*11*). Many studies suggest that a higher proportion of IPF is associated with worse clinical outcomes in patients with cardiovascular diseases and in acute coronary syndromes (*7*, *9*). Immature platelets are metabolically and enzymatically more active than mature ones, having a greater prothrombotic activity. In consequence, some authors have hypothesized that IPF values may guide antiplatelet therapy as a marker of platelet turnover. Due to the major prothrombotic activity, a higher proportion of IPF could be associated with an attenuated response to antiplatelet drugs (*12*).

Recent studies suggest that IPF can provide clinical information about inflammatory conditions (*8*). In patients with sepsis, the activity of the bone marrow may also result in the release of an increased concentration of myeloid precursors and earlier platelets in peripheral blood. The mechanism inducing the increase in the production of IPF in septic patients could be related to the increase in cytokine production which occurs due to the inflammatory state (*13*).

At the beginning, IPF was measured by means of staining for RNA assessed by flow cytometry. However, this method had several limitations, such as being very time-consuming, having a high cost and a lack of standardization. Nowadays, automated IPF measurement is integrated into routine haematology analysers. For example, the newly developed Sysmex XN-series haematology analyser adopts a new platelet fluorescent channel (PLT-F) to measure IPF.

Despite the above-mentioned clinical benefits, the provision of reliable reference intervals for IPF is necessary to evaluate its usefulness and avoid misdiagnoses. The International Federation of Clinical Chemistry (IFCC) and Clinical and Laboratory Standard Institute (CLSI) recommend that all clinical laboratories should develop their own biological reference intervals (*14*). However, few laboratories currently follow this recommendation because of its difficulty, great time consumption and high costs.

The reference interval for IPF in a Spanish population employing the Sysmex XN-series have not yet been studied. According to previous studies, IPF values are highly dependent on the analyser used and also on ethnic background, but usually no differences between genders are found (*15*, *16*). Given these facts, our hypothesis is that reference intervals performed using the XE-series may not be appropriate for the XN-series. Moreover, the previously reported reference intervals established using the XN-series were developed in an Asian population, hence they too may not be valid for the Spanish population.

For all these reasons, the aim of the present work was to define 95% reference limits for IPF using the Sysmex XN-2000 in an adult Spanish population following CLSI guidelines.

## Materials and methods

### Subjects

The study was performed at Bellvitge University Hospital (Barcelona, Spain) which is a 700-bed teaching hospital specialized in adult patient care. The clinical laboratory is accredited according to the International Standard ISO 15189.

A total of 164 Caucasian volunteers from Spain were enrolled in this study from December 2015 to September 2016. Inclusion criteria for individuals were the following: ≥ 16 years old and apparently healthy adults. Exclusion criteria were the following: any acute or chronic disease, pregnancy, donating blood or receiving a blood transfusion in the past year, history of alcoholism, smoking > 20 cigarettes per day and taking any drug known to affect the platelet number or activity (*e.g.* salicylates). Moreover, to exclude unhealthy individuals, blood, serum and plasma were obtained from each volunteer to assess the complete blood count (CBC), biomarkers for liver and renal function, iron status and coagulation. Subjects were excluded if any laboratory results were outside the reference intervals used in our clinical laboratory. This study was approved by the Clinical Research Ethics Committee of Bellvitge University Hospital. A completed questionnaire and written informed consent were obtained from each individual.

### Methods

Blood samples were collected in 4 mL tubes containing ethylenediaminetetraacetic acid (K_3_EDTA) (Vaccuette®, Greiner Bio One®, Kremsmünster, Austria) and stored at room temperature before IPF measurement. All samples were analysed within 2–4 hours of collection. Complete blood count and IPF were measured using a Sysmex XN-2000 (Sysmex, Kobe, Japan) according to the manufacturer’s recommendation. The Sysmex XN-series adopts fluorescent flow cytometry with semiconductor diode laser to measure IPF. Briefly, oxazine fluorescent dyes penetrate the platelets and stain the RNA. The stained platelets are passed through a semiconductor diode laser beam and the resulting forward scatter light (volume) and fluorescence intensity (mRNA content) are measured. The mature and immature platelets are identified on the basis of their fluorescence intensity. The IPF is expressed as a proportional value (%-IPF) of the total platelet count to indicate the rate of platelet production.

Throughout the study period, three levels of quality control material (QC-XN-CHECK levels 1, 2 and 3; Streck Laboratories Inc., Omaha, USA) were performed for all routine parameters, fluorescent platelets count and IPF, to control the performance of the overall procedure and the instrument under use.

Within- and between-day imprecision and relative bias were estimated for IPF using the lower QC material. The IPF conventional value was assigned by the manufacturer. The desirable limits for within- and between-day analytical variations (CV_A_) and relative bias were defined by Bouro *et al.* (*17*). The within- and between-day imprecision obtained were 1.6 and 2.6%, respectively (desirable limit: 3.9%) and - 2.6% for relative bias (desirable limit: 11.9%). The metrological characteristics for IPF measurement did not exceed the desirable limits reported (*17*).

In addition, in order to exclude potentially unhealthy individuals, serum was collected in serum gel tubes and plasma in sodium citrate solution tubes (Vacuette®, Greiner Bio-One GmbH®, Kremsmünster, Austria) to measure all biochemical parameters on a Cobas 8000 (Roche Diagnostics®, Basel, Switzerland) and coagulation parameters on an ACL-TOP 500 CTS (Instrumentation Laboratory Company®, Bedford, USA), respectively.

### Statistical analysis

All calculations to determine reference intervals were based on the CLSI guidelines (document EP28-A3c) (*14*).

Outliers were detected and removed using the adjusted Tukey’s test. The distribution plot was performed to visually inspect the data. The Kolmogorov–Smirnov test was used to assess the normality of distribution of the data.

According to the normality of the data, appropriate measures and statistical tests were used. Normally distributed data were expressed as mean ± standard deviation (SD). The non-normally distributed data were expressed as median and interquartile range (IQR). Significance of between-group differences was tested by the parametric independent t-test and non-parametric Mann-Whitney test.

The IPF median values in males and females were compared using the Mann–Whitney U-test. When the results did not warrant partitioning, data were combined to calculate the reference interval.

The reference interval was calculated using a non-parametric method for lower and upper reference limits as 2.5% and 97.5% of the reference distribution. The 90% confidence intervals (CI) were calculated for lower and upper reference limits.

The values P < 0.05 were considered statistically significant. Statistical analysis was performed using STATA® version 14 (StataCorp LP, Texas, USA).

## Results

Blood samples were obtained from all eligible participants (N = 164). Three subjects were excluded due to abnormal total platelet count (in-house reference values: 149–303 and 153–368 x10^9^/L for males and females, respectively), and one subject due to low haemoglobin concentration (WHO reference values: > 130 and > 120 g/L for male and females, respectively). In total, four participants did not fulfill all the inclusion criteria and were removed from further analysis. In addition, seven extreme values were detected and discarded as outliers. In summary, 153 subjects, comprising 73 males and 80 females, were enrolled for calculating the reference intervals for IPF on a Sysmex XN-2000.

The median age (min, max) of the total group was 37 (19–76) years. The median age of males and females was 31 (19–76) and 41 (20–68) years, respectively (P = 0.074).

**Figure 1:**
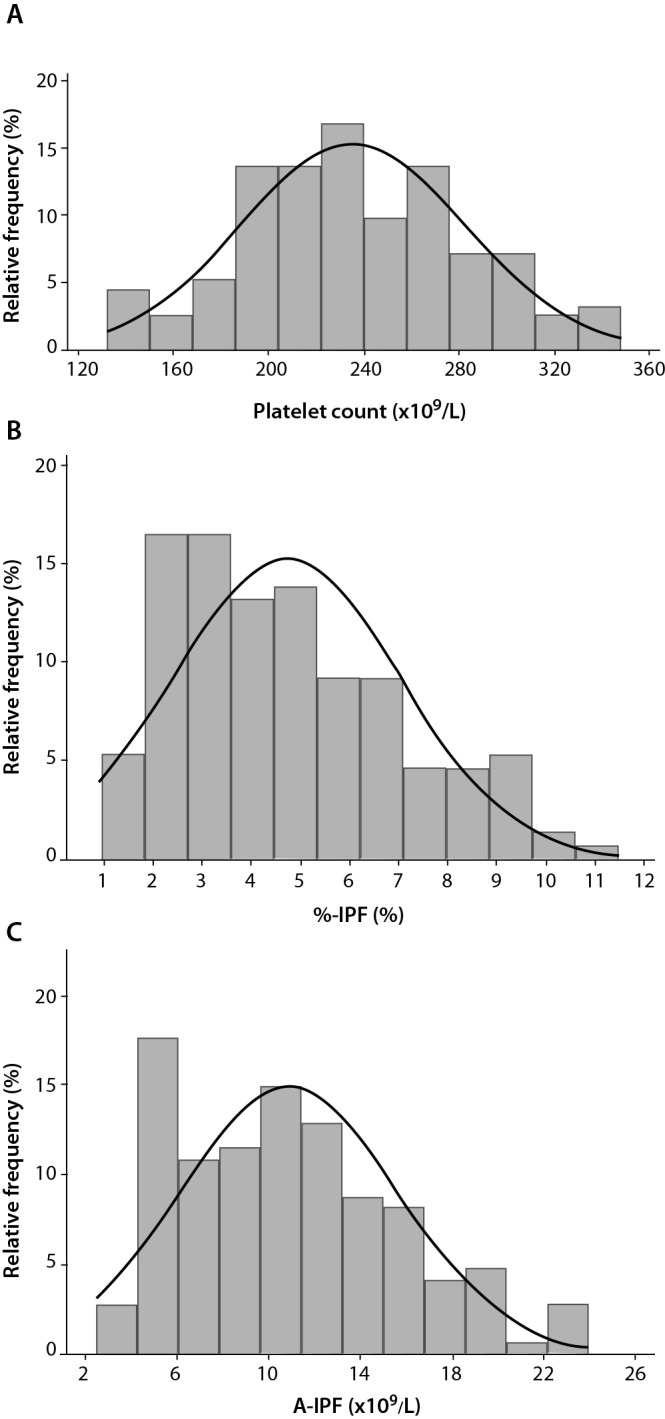
(A) Distribution of platelet count. (B) Distribution of immature platelet fraction (IPF). (C) Distribution of absolute immature platelet count (A-IPF) in reference individuals (N = 153).

The distribution of the complete reference data for total platelet count, %-IPF and A-IPF, are presented in a scatter plot (Figure 1). The distribution for platelet count was Gaussian (P = 0.435) and for %-IPF and A-IPF was non-Gaussian (P < 0.001 and P = 0.002, respectively), showing left-skewed histograms.

Data for total platelet count, %-IPF and A-IPF from healthy individuals are presented in Table 1. No statistical differences were observed between genders in any of these three parameters.

**Table 1:** Platelet count, immature platelet fraction and absolute number from healthy individuals.

	**Total** **(N = 153)**	**Male** **(N = 73)**	**Female** **(N = 80)**	**P value**
**Platelet count (x10^9^/L)**	235 ± 47	230 ± 43	240 ± 50	0.158*
**IPF (%)**	4.3 (2.9-6.2)	4.1 (2.6-5.9)	4.7 (3.3-6.4)	0.101^†^
**A-IPF (x10^9^/L)**	10.23 (6.81-14.16)	9.46 (6.20-13.24)	11.02 (7.59-15.05)	0.071^†^
Data are expressed as a mean ± standard deviation for platelet count, and as a median (IQR) for IPF and A-IPF. *Differences between men and women were tested using the t-student's test. ^†^Differences between men and women were tested using the Mann-Whitney U test. IPF - immature platelet fraction. A-IPF - absolute immature platelet count.				

Finally, the reference interval and 90% corresponding confidence intervals (90%CI) for IPF in total individuals were 1.6 (90%CI: 1.5–1.8) – 9.6 (90%CI: 9.3–11.5)%.

## Discussion

Nowadays, automated haematology analysers have been improved to enable the measurement of immature platelets. In the present research, we show, for the first time, the reference interval for IPF in a Spanish cohort using the Sysmex XN-2000. The reference values estimated were 1.6–9.6%, independent of gender.

To promote the clinical use of IPF, establishing reliable reference interval should be mandatory. To the best of our knowledge, no data are available for IPF adult reference intervals in a Spanish or southern European population in Sysmex XN-series analysers. Therefore, we aimed to define 95% reference limits for IPF and, whenever relevant, establish separate reference intervals according to gender. On the basis of our results and in accordance with previously reported data, we did not confirm statistically significant differences of reference intervals for male *versus* female for IPF (*2*, *15*, *18*, *19*). Thus, we assume that a gender-specific reference interval would be unnecessary.

The upper reference limit established in this study is somewhat higher than in previously published papers, ranging from 3.2 up to 7.1% (*2*, *15*, *20*). One can remark that all of these studies were performed with a Sysmex XE-series analyser. In comparison with the Sysmex XE, the Sysmex XN seems to be higher in %IPF. This fact was also described by Ko *et al.* (*16*). According to Van der Linden *et al.* and Ko *et al*., the higher reference intervals of IPF obtained with the XN compared with the XE series may reflect the fact that both analysers have different metrological characteristics and that IPF measured on the XN series has a better precision and accuracy than when measured on the XE series (*5*, *16*). With the introduction of the XN series, the principles, channel, reagent and algorithm to detect IPF have been changed. Given these differences, IPF results will be more specific and this suggests the necessity of new reference intervals for IPF.

Nevertheless, in contrast with our hypothesis, the reference intervals reported previously in a Korean population using the Sysmex XN are similar to the current study, between 1 and 9% (*16*). These results suggest that IPF values seem to be more affected by the analyser used than the ethnic background of the individuals.

This study was, however, limited by the small number of enrolled reference individuals. Nevertheless, it was enough to establish the reference intervals following the CLSI guidelines. Also, it has to be taken into account that age differences have not been assessed in the establishment of reference intervals. Despite this, previous studies indicate that no differences were found in IPF results when stratifying by age (*18*, *19*). Finally, this study did not establish the reference intervals for A-IPF. However, the clinical utilities of immature platelets, expressed as a proportional value (%-IPF), are more widely reported in the literature than A-IPF.

In conclusion and to the best of our knowledge, we have generated the first report on reference intervals of IPF in a Spanish population using the Sysmex XN-2000 haematology analyser. The established reference interval for IPF was 1.6–9.6%, independent of gender. These results may be of benefit to the laboratories using the Sysmex XN-series analyser, making an accurate interpretation of patients’ results much easier.
